# High Plasma Levels of sTNF-R1 and CCL11 Are Related to CD4+ T-Cells Fall in Human Immunodeficiency Virus Elite Controllers With a Sustained Virologic Control

**DOI:** 10.3389/fimmu.2018.01399

**Published:** 2018-06-18

**Authors:** Mónica Gutiérrez-Rivas, María Ángeles Jiménez-Sousa, Norma Rallón, José Luis Jiménez, Clara Restrepo, Agathe León, Marta Montero-Alonso, Juan González-García, María Ángeles Muñoz-Fernández, José Miguel Benito, Salvador Resino, on Behalf of ECRIS Integrated in the Spanish AIDS Research Network

**Affiliations:** ^1^Unidad de Infección Viral e Inmunidad, Centro Nacional de Microbiología, Instituto de Salud Carlos III, Majadahonda, Spain; ^2^Instituto de Investigación Sanitaria Fundación Jiménez Díaz, Universidad Autónoma de Madrid (IIS-FJD, UAM), Madrid, Spain; ^3^Hospital Universitario Rey Juan Carlos, Móstoles, Spain; ^4^Plataforma de Laboratorio, Hospital General Universitario “Gregorio Marañón”, Madrid, Spain; ^5^Sección Inmunología, Laboratory InmunoBiología Molecular, Hospital General Universitario “Gregorio Marañón”, Madrid, Spain; ^6^Servicio de Enfermedades Infecciosas, Hospital Clinic-IDIBAPS, Barcelona, Spain; ^7^Unidad de Enfermedades Infecciosas, Hospital Universitario y Politécnico “La Fe”, Valencia, Spain; ^8^Servicio de Medicina Interna, Hospital Universitario “La Paz”, Madrid, Spain; ^9^Instituto de Investigación Sanitaria del Gregorio Marañón, Madrid, Spain

**Keywords:** human immunodeficiency virus, elite controllers, inflammation, plasma biomarkers, acquired immune deficiency syndrome, progression

## Abstract

Our aim was to analyze the relationship between plasma inflammatory biomarkers and CD4+ T-cells evolution in human immunodeficiency virus (HIV) elite controllers (HIV-ECs) with a suppressed viremia. We carried out a retrospective study in 30 HIV-ECs classified into two groups: those showing no significant loss of CD4+ T-cells during the observation period (stable CD4+, *n* = 19) and those showing a significant decrease of CD4+ T-cells (decline CD4+, *n* = 11). Baseline plasma biomarkers were measured using a multiplex immunoassay: sTNF-R1, TRAIL, sFas (APO), sFasL, TNF-α, TNF-β, IL-8, IL-18, IL-6, IL-10, IP-10, MCP-1, MIP-1α, MIP-1β, RANTES, SDF1α, GRO-α, and CCL11. Baseline levels of sTNF-R1 and CCL11 and sTNF-R1/TNF-α ratio correlated with the slope of CD4+ T-cells (cells/μl/year) during follow-up [*r* = −0.370 (*p* = 0.043), *r* = −0.314 (*p* = 0.091), and *r* = −0.381 (*p* = 0.038); respectively]. HIV-ECs with declining CD4+ T-cells had higher baseline plasma levels of sTNF-R1 [1,500.7 (555.7; 2,060.7) pg/ml vs. 450.8 (227.9; 1,263.9) pg/ml; *p* = 0.018] and CCL11 [29.8 (23.5; 54.9) vs. 19.2 (17.8; 29.9) pg/ml; *p* = 0.041], and sTNF-R1/TNF-α ratio [84.7 (33.2; 124.2) vs. 25.9 (16.3; 75.1); *p* = 0.012] than HIV-1 ECs with stable CD4+ T-cells. The area under the receiver operating characteristic (ROC) curve [area under ROC curve (AUROC)] were 0.758 ± 0.093 (sTNF-R1), 0.727 ± 0.096 (CCL11), and 0.777 ± 0.087 (sTNF-R1/TNF-α). The cut-off of 75th percentile (high values) for these biomarkers had 71.4% positive predictive value and 73.9% negative predictive value for anticipating the evolution of CD4+ T-cells. In conclusion, the loss of CD4+ T-cells in HIV-ECs was associated with higher levels of two plasma inflammatory biomarkers (sTNF-R1 and CCL11), which were also reasonably accurate for the prediction of the CD4+ T-cells loss.

## Introduction

CD4+ T-cells are the major target for human immunodeficiency virus (HIV); therefore, a gradual CD4+ T-cells count decline and progression to acquired immune deficiency syndrome (AIDS) are normally observed during untreated HIV-infection (1). In contrast, elite controllers [HIV elite controllers (HIV-ECs)] are a subgroup of antiretroviral treatment-naïve HIV-infected patients (<1%) that naturally suppress HIV viremia (generally <50 copies/ml) with relative CD4+ T-cells preservation and delayed AIDS progression, but a persistent low-level HIV replication is detectable by ultrasensitive assays (2). Moreover, there is substantial interindividual variability in the rate and extent of progression to AIDS in HIV-ECs (3). Thus, most patients have elevated CD4+ T-cells count, stable CD4+ T-cell trajectories, and more favorable clinical outcomes compared with viremic patients; but a subgroup of HIV-ECs may progress to AIDS with CD4+ T-cells decline and/or loss of virologic control (4). In this regard, HIV-ECs showed lower marker levels of inflammatory and immune activation than HIV viremic controllers (HIV-VCs) (5, 6).

There is evidence of ongoing inflammation, bacterial translocation, T-cells activation, and CD4+ T-cells depletion on HIV-ECs, suggesting that this natural long-term HIV control may have an immunologic and clinical cost (3, 7–10). Chronic immune activation associated with HIV infection may lead to fibrosis in the lymphoid tissues, where HIV replicates, for dramatically altering the structure and function, giving rise to a progressive loss of CD4+ T-cells, particularly in the naive T-cells subset (11, 12). In this regard, HIV-ECs also have significant lymphoid tissue fibrosis and CD4+ T-cells depletion at lymphoid tissue, similar to HIV non-controllers (13).

The role of immune activation in HIV-1 pathogenesis has been broadly analyzed, but immune activation in HIV disease progression is not well characterized in HIV-ECs. The aim of our study was to analyze the relationship between plasma inflammatory biomarkers and CD4+ T-cells evolution in HIV-ECs with a suppressed viremia.

## Materials and Methods

### Patients

We carried out a retrospective study with 30 HIV-ECs from the cohort of HIV controllers of the Spanish AIDS Research Network (ECRIS cohort), launched in 2013. ECRIS is a multicentre cohort of HIV controller patients whose data come from the long-term non-progressors cohort, the cohort of the Spanish AIDS Research Network (CoRIS) (3), and from different clinical centers (see Appendix S1 in Supplementary Material). To be included in ECRIS cohort, EC patients were defined as asymptomatic individuals with at least three consecutive plasma HIV viral load (pVL) determinations below the detection limit (pVL < 50 copies/ml) during a period of 12 months in the absence of any antiretroviral therapy. Characteristics of the ECRIS cohort have been described in detail elsewhere (3).

The study protocol was approved by the Institutional Review Boards of the participating hospitals. All patients gave written informed consent in accordance with the Declaration of Helsinki.

Starting from this cohort, we selected HIV-ECs that had long-term control of HIV replication (minimum of 3 years), during which the evolution of their CD4+ T-cells levels was evaluated and with baseline plasma sample available for study. For each patient, a slope of CD4+ T-cells was calculated by linear regression models with all CD4+ T-cells count throughout the time of each patient’s follow-up. Based on this CD4+ T-cells slope, the study patients were classified into two groups: those who showed no significant (*p* ≥ 0.05) loss of CD4+ T-cells during the observation period (stable CD4+, *n* = 19) and those who had a significant decrease (*p* < 0.05) in the levels of CD4+ T-cells (decline CD4+, *n* = 11).

The clinical and epidemiological data were provided by ECRIS cohort. Furthermore, samples from patients were kindly provided by the Spanish HIV HGM BioBank integrated in the Spanish AIDS Research Network (RIS) (14). Samples were processed following current procedures and were frozen at −80°C immediately after their reception (14). The first biological sample available after the inclusion of each patient in the cohort was used for laboratory assays.

### Multiplex ELISA

Multiplex kits (ProcartaPlex™ Multiplex Immunoassay; Affymetrix eBioscience, San Diego, CA, USA) were used to specifically evaluate plasma biomarkers according to the manufacturer’s specifications using the Luminex 100™ analyzer (Luminex Corporation, Austin, TX, USA). The kits used were Affymetrix Human Chemokine 9plex panel [Eotaxin (CCL11), GRO-α (KC/CXCL1), IL-8 (CXCL8), IP-10 (CXCL10), MCP-1 (CCL2), MIP-α (CCL3), MIP-β (CCL4), RANTES (CCL5), and SDF-1α (CXCL12)], ProcartaPlex Simplex [TNF-β, IL-18, sFas (APO), sTNF-RI, TRAIL, and sFasL], and ProcartaPlex High Sensitivity (IL-6, IL-10, and TNF-α).

### Statistical Analysis

The statistical analysis was performed with the Statistical Package for the Social Sciences (SPSS) 21.0 (SPSS Inc., Chicago, IL, USA). Statistical significance was defined as *p* < 0.05. All *p*-values were two-tailed. Values were expressed as absolute number (percentage) and median (25th; 75th percentile).

Categorical data and proportions were analyzed using the chi-squared test or Fisher’s exact test (when expected values were below 5). Mann–Whitney *U* test was used to compare data among independent groups (stable CD4+ vs. decline CD4+). Correlation was analyzed using the Pearson correlation coefficient.

We used the logistic regression analysis to test the association between levels of plasma inflammatory biomarkers and the two study groups according to the evolution of CD4+ T-cells slope (stable CD4+ vs. decline CD4+). When a significant association value was obtained, we saved the value of predicted probability for each patient and we evaluated the diagnostic performance of plasma biomarkers for predicting the evolution of CD4+ T-cells using the receiver operating characteristic (ROC) curve. We also calculated the sensitivity (Se), specificity (Sp), positive predictive value (PPV), and negative predictive value (NPV) for analyzing the cut-off point of the highest values (75th percentile).

## Results

### Study Population

The characteristics of the 30 HIV-ECs with sustained viremia control (19 with stable CD4+ T cells count and 11 with decreasing CD4+ T cells count) are shown in Table 1. Overall, the median age was 41 years, 43.3% were males, and more than 40% were coinfected with hepatitis C virus (HCV). The CD4+ T-cells count at the beginning of the follow-up was 959 cells/μl and the time of follow-up was 12 years. Several characteristics were similar in the two groups of HIV-ECs, except for the slope of CD4+ T-cells, the CD4+ T-cell count at the end of follow-up, and a slight difference in time of follow-up.

**Table 1:** Characteristics of HIV-infected patients included in the study.

Characteristic	All patients	Stable CD4+	Decline CD4+	*p*-Value
No.	30	19	11	
Age (years)	41 (36; 47)	40 (33; 46)	42 (38; 48)	0.324
Gender (males)	13 (43.3%)	5 (26.3%)	7 (63.6%)	0.104
Presence of HCV coinfection	13 (43.3%)	9 (57.9%)	4 (54.5%)	0.842
CD4+ T-cell count (cells/μl) at beginning of follow-up	959 (793; 1,147)	856 (675; 1,145)	1,053 (850; 1,153)	0.130
Follow-up (years)	12 (8.5; 12.3)	12 (11; 13)	11 (6; 12)	0.047
CD4+ T-cell slope (cells/μl/year) during follow-up	−2.5 (−35; 13)	8 (−2; 54)	−47 (−86; −30)	<0.0001
CD4+ T-cell count (cells/μl) at the end of follow-up	816 (632; 1,110)	1,005 (725; 1,190)	633 (503; 978)	0.016
Number of HIV-RNA blips (pVL > 50 copies/ml)	1 (0; 2)	1 (0; 2)	1 (0; 2)	0.497

*Values were expressed as median (percentile 25; percentile 75) for quantitative variables, and as absolute number (percentage) for qualitative variables*.

*HCV, hepatitis C virus; HIV, human immunodeficiency virus*.

### Relationship Analysis

**Figure 1:**
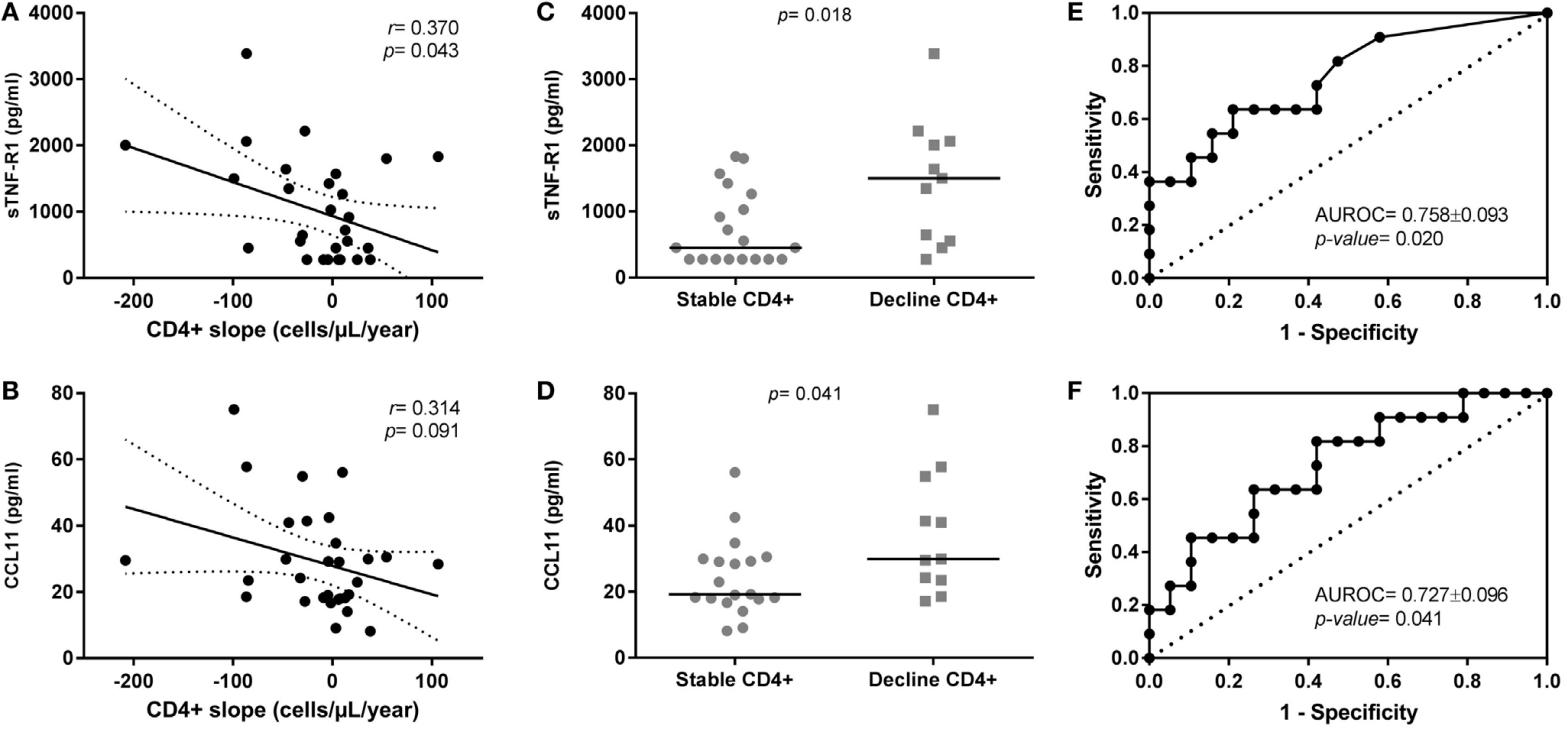
Relationship of sTNF-R1 and CCL11 plasma levels with CD4+ T-cells evolution in human immunodeficiency virus (HIV) elite controllers (HIV-ECs) with sustained virologic control. **(A,B)** Show dot-plot graphs of sTNF-R1 and CCL11 vs. CD4+ slope in the whole population of HIV-ECs; **(C,D)** show levels of sTNF-R1 and CCL11 in the two groups of HIV-ECs (stable CD4 and decline CD4); **(E,F)** show the receiver operating characteristic curves for sTNF-R1 **(E)** and CCL11 **(F)** as predictors of CD4+ decline in HIV-ECs.

We found baseline plasma levels of sTNF-R1 (Figure 1A) and CCL11 (Figure 1B) correlated with the slope of CD4+ T-cells (cells/μl/year) during the follow-up [*r* = −0.370 (*p* = 0.043) and *r* = −0.314 (*p* = 0.091), respectively]. Besides, sTNF-R1/TNF-α ratio also showed a significant negative correlation with the slope of CD4+ T-cells [*r* = −0.381 (*p* = 0.038)]. An absence of correlation was found between sTNF-R1 and CCL11 [*r* = 0.327 (*p* = 0.078)].

### Diagnostic Performance

We analyzed the differences between groups and the diagnostic performance of plasma biomarkers on the evolution of CD4+ T cells count (full data in Table S1 in Supplementary Material).

We found that HIV-ECs with decline CD4+ T-cells had higher baseline plasma levels of sTNF-R1 [1,500.7 (555.7; 2,060.7) pg/ml vs. 450.8 (227.9; 1,263.9) pg/ml; *p* = 0.018] and CCL11 [29.8 (23.5; 54.9) vs. 19.2 (17.8; 29.9) pg/ml; *p* = 0.041] than HIV-1 ECs with stable CD4+ T-cells (Figures 1C,D; respectively). Moreover, we found that the baseline ratio of sTNF-R1/TNF-α was higher in HIV-ECs with decline CD4+ T-cells [84.7 (33.2; 124.2) vs. 25.9 (16.3; 75.1); *p* = 0.012] than HIV-1 ECs with stable CD4+ T-cells.

The area under receiver operating characteristic curve (AUROC) of sTNF-R1 was 0.758 ± 0.093 (*p* = 0.020) (Figure 1E) and of CCL11 was 0.727 ± 0.096 (*p* = 0.041) (Figure 1F) for predicting the CD4+ T-cells loss in HIV-ECs. The AUROC was 0.789 ± 0.084 (*p* = 0.009) for the two molecules together (sTNF-R1 and CCL11). That is, there was practically no improvement in the value of AUROC with respect to each biomarker separately. Moreover, we found that the AUROC for baseline ratio of sTNF-R1/TNF-α was 0.777 ± 0.087 (*p* = 0.013).

Using the cut-off of 75th percentile for sTNF-R1 (1,589.28 pg/ml), CCL11 (36.31 pg/ml), and sTNF-R1/TNF-α (91.1), these three biomarkers showed similar values: the Se was 45.5%, the Sp was 89.5%, the PPV was 71.4%, and the NPV was 73.9%.

## Discussion

In this study, we found a relationship of two plasma inflammatory biomarkers (sTNF-R1 and CCL11) with the CD4+ slopes during the long-term control of HIV replication in our cohort of HIV-ECs.

The immune activation is a hallmark of HIV-1 disease and the role of immune activation in HIV-1 pathogenesis and immune dysfunction is recognized (15). Previous reports have characterized immune activation during HIV-1 infection and attempted to determine the relationship with CD4+ T-cells loss and AIDS progression (16, 17). Additionally, HIV-ECs maintain control of plasma HIV viremia, but have evidence of an activated innate immune response (7). In a recent article, a subtle decline of % CD4+ T-cells was observed in HIV-VCs, but not in HIV-ECs, which was associated with higher plasma levels of proinflammatory cytokines (5). Moreover, Pernas et al. showed that RANTES, and to a lesser extent CCL24 (eotaxin-2), could be biomarkers of EC transition from natural virological control to the loss of virological control (18), since higher levels of chemokines could reflect higher low-level residual HIV replication (19). The mechanisms underlying CD4+ T-cells loss and AIDS progression in HIV-ECs are poorly understood and are likely to be multifactorial. The viral replication is not completely suppressed in HIV-ECs and persistent low-level of HIV replication in HIV-ECs may be responsible for the increase of T-cell activation and systemic inflammation (2, 20), which have been related to fall of CD4+ T-cells count and AIDS progression among HIV-infected patients (21, 22). HIV-ECs have also elevated markers of microbial translocation compared with either the HIV-suppressed or the uninfected groups, which may induce immune activation and systemic inflammation (10), and antiretroviral therapy diminished levels of residual viremia and T-cell activation (23).

In our study, higher values of plasma CCL11 were linked to CD4+ T-cells loss; while low values of CCL11 were associated with stable CD4+ T-cells count during the long-term follow-up. In previous studies, plasma levels of CCL11 have been related to a worse virological outcome during primary HIV-1 infection and post analytical treatment interruption (24). Moreover, CCL11 has also previously been associated with more rapid CD4 loss below 350 cells/μl during acute HIV-1 infection (25). It indicates that the influence of CCL11 on the risk of CD4 loss occurs also in other HIV-infected populations. However, it is important to note that other studies did not find any association between CCL11 and HIV disease progression (viral load and CD4 count) (26). CCL11 is a chemokine that selectively attracts lymphocytes through chemokine receptors (CCR3 and CCR5), which are also HIV co-receptors (27). Thus, CCL11 may have an anti-HIV effect through its binding capacity to one of HIV coreceptors (27–29), but also its chemotactic ability on CD4+ T-cells may also serve to recruit targets for HIV infection (30). In fact, elevated CCL11 levels in placental plasma were associated with *in utero* mother-to-child transmission (31).

TNF-α is an important mediator of immune activation driven by high levels of HIV replication, which is linked to CD4+ T-cells loss and HIV-1 disease progression (32). TNF-R1 mediates most of the cellular responses induced by TNF-α and may circulate in soluble form after its proteolytic cleavage (sTNF-R1), binding to the circulating TNF-α and inhibiting its activity (negative feed-back) (33). In our study, higher plasma values of sTNF-R1 and sTNF-R1/TNF-α ratio were associated with falls of CD4+ T-cells in HIV-ECs; while values of TNF-α did not correlate with CD4+ T-cells slopes. This would indicate that the biomarker actually associated with the loss of CD4+ T-cells is sTNF-R1, perhaps because plasma TNF-α could be neutralized by sTNF-R1. In any case, higher values of sTNF-R1 would indicate higher levels of inflammation and immune activation, which impact both viral replication and viral persistence, and CD4+ T-cells loss (34). Besides, higher levels of sTNF-R1 have been related to a poor immune response to successful antiretroviral therapy (35) and poor response to hepatitis B virus vaccine (36) in HIV-infected patients. Additionally, similarly to CCL11, the association between higher TNF-R1 level and poor CD4+ T cell recovery has been previously described, indicating that this finding could be also applicable to other HIV-infected populations (35).

Our data showed that plasma sTNF-R1 and CCL11 values were accurate for the prediction of the CD4+ T-cells loss, since the AUROC value was close to 0.75, which supports its acceptable ability to discriminate HIV-ECs in risk of losing CD4+ T-cells. Furthermore, the cut-offs evaluated in our study showed values of NPV and PPV higher than 70%, which could be acceptable for excluding a CD4+ T-cells loss or for predicting it; respectively. Thus, these inflammatory biomarkers could help manage HIV-ECs in the clinical setting.

Finally, several aspects have to be taken into account for the correct interpretation of the results. First, the retrospective nature of design might introduce biases in the analysis and a lack of uniformity. Second, this is a preliminary study with a limited number of patients, which limits for achieving statistically significant differences. It is remarkable that, among 18 biomarkers, only two were related to the loss of CD4+ T-cells in HIV-ECs. With a higher number of subjects included in this study, possibly more biomarkers would be identified. Third, the high number of biomarkers analyzed, coupled to the low number of patients included, penalize for achieving significant differences when we adjusted the *p*-values by multiple comparisons (Bonferroni correction). Fourth, the different definitions of HIV-ECs could influence on the results obtained and the compatibility with other studies.

In conclusion, the loss of CD4+ T-cells in HIV-ECs was associated with higher levels of two plasma inflammatory biomarkers (sTNF-R1 and CCL11), which were also reasonably accurate for the prediction of the CD4+ T-cells loss. Further analysis involving large numbers of patients in independent cohorts are needed to corroborate these associations and to understand the mechanism leading to increased sTNF-R1 and CCL11 production, as well as to determine any long-term impact on immune dysfunction.

## Ethics Statement

The study protocol was approved by the Institutional Review Boards of the participating hospitals. All subjects gave written informed consent in accordance with the Declaration of Helsinki.

## Author Contributions

Conceptualization: NR, JB, and SR. Resources and data curation: NR, JB, CR, AL, JJ, MM-A, JG-G, MM-F. Investigation and writing—original draft preparation: MG-R, MJ-S, and SR. Formal analysis: SR. Writing—review and editing: NR and JB. Visualization, supervision, and funding acquisition: NR, JB, and SR.

## Conflict of Interest Statement

The authors declare that the research was conducted without any commercial or financial relationships that could be construed as a potential conflict of interest.
